# Functional characterization of BmOVOs in silkworm, *Bombyx mori*

**DOI:** 10.1186/s12864-019-5697-y

**Published:** 2019-05-06

**Authors:** Min Zhu, Xiaolong Hu, Zi Liang, Mengsheng Jiang, Renyu Xue, Yongchang Gong, Xing Zhang, Guangli Cao, Chengliang Gong

**Affiliations:** 1 School of Biology and Basic Medical Sciences, Soochow University, No.199 Ren’ai Road, Dushu Lake Higher Education Town, Suzhou Industrial Park, Suzhou, Jiangsu 215123 People’s Republic of China; 2 National Engineering Laboratory for Modern Silk, Soochow University, Suzhou, 215123 Jiangsu China; 3 Agricultural Biotechnology Research Institute, Agricultural biotechnology and Ecological Research Institute, Soochow University, Suzhou, 215123 China

**Keywords:** *Bombyx mori*, BmOVO, Promoter activity, Ovarian tumor gene, Tal-like

## Abstract

**Background:**

In our previous study, we identified four isoforms of the *Bmovo* gene, *Bmovo-1*, *Bmovo-2*, *Bmovo-3* and *Bmovo-4* from the silkworm ovary and verified that ovarian development was regulated by the BmOVO proteins. Results: To understand the regulatory mechanisms of ovarian development, the regulation of four BmOVO isoforms on the *B. mori ovarian tumor* (*Bmotu*) promoter activity was investigated with luciferase reporter assays. The results showed the *Bmotu* promoter activity was positively regulated by BmOVO-1, BmOVO-2, BmOVO-3 and BmOVO-4 in a dose-dependent manner, of which BmOVO-2 had the highest transcriptional activation. However, the first (A1) and third acidic domains (A3) at the N-terminus of BmOVO-1 are transcriptional repression domains, while the fourth (A4) and fifth acidic domains (A5) are transcriptional activation domains. A recombinant BmOVO zinc-finger domain was found to bind to the GTACCGTTGTA sequence located at the *Bmotu* promoter. Furthermore, the *Bmotu* promoter activity was negatively regulated by ‘Tal-like’ peptide, which can trigger BmOVO-1 degradation at the N-terminus.

**Conclusions:**

These results will help us to further understand the regulatory mechanisms of BmOVO isoforms on *Bmotu* promoter activity and ovarian development in the silkworm.

**Electronic supplementary material:**

The online version of this article (10.1186/s12864-019-5697-y) contains supplementary material, which is available to authorized users.

## Background

OVO proteins belong to members of the zinc finger protein family and serve as transcription factors that regulate gene expression in various differentiation processes. OVOs not only participate in the development of the neural tube and germ cells, but also play an important role in eye regeneration and the maintenance of the eye in adult planarians [1–6]. In *Drosophila*, the *ovo* locus encodes three isoforms, Svb, OVO-A and OVO-B [7]. They share four identical C_2_H_2_ zinc fingers at their carboxyl (C) termini, which bind to specific DNA sequences [8], but the amino (N) terminus of these isoforms varies. Svb has a role in both larval and adult trichome development and has been detected in the embryonic ventral epidermis [9, 10]. OVO-A and OVO-B are only expressed in germ cells and contribute to ovarian development. OVO-A and OVO-B have opposite regulatory activities that are required for female germline development and oogenesis. OVO-A is considered to be a transcriptional repressor, while OVO-B has been identified as an activator [7]. O*varian tumor* (*otu*), a target gene of OVO proteins, is essential for the viability and differentiation of the female germline in *Drosophila* [11]. OVO-B positively regulates *otu* gene expression while OVO-A represses it [8, 12]. Moreover, OVO-B also plays an important role in epidermis development when it is expressed ectopic in yeast. [13].

The silkworm (*Bombyx mori*), a model species of Lepidoptera, has many genes which are homologous to those of *Drosophila*. In our previous studies, four alternatively spliced isoforms of the *B. mori ovo* (*Bmovo*) gene, which have been designated as *Bmovo-1*, *Bmovo-2*, *Bmovo-3* and *Bmovo-4* according to their deduced molecular weights from large to small, were identified in the ovary. Sequence comparisons showed that 203 amino acid residues were conserved at the C-terminus among BmOVO-1, BmOVO-2, BmOVO-3 and BmOVO-4, and four common C_2_H_2_ zinc fingers were found at the C-termini of BmOVO-1, BmOVO-2 and BmOVO-3, while only one was found in BmOVO-4, suggesting that BmOVOs may be transcription factors [14]. Various effectors domains at the N-termini of BmOVOs may lead to differences in their functions, and our previous study showed that *Bmovo-1* overexpression in silkworm ovaries might promote anabolism for ovarian development [15]. However, the roles of the effector domains of BmOVOs in the regulation of gene expression are still unknown.

Sex determination and differentiation of *Bombyx mori* has always been one of the important research directions in silkworm industry. In the long-term practice of sericulture, it has been found that the cocoon silk production capacity of male silkworm is generally higher than that of female silkworm, which is generally believed to be due to the fact that male silkworm is strong, does not need to consume extra nutrients for egg making and has high leaf silk conversion rate. *B. mori ovarian tumor* (*Bmotu*) is homologous with *Drosophila otu*, which is essential for female germline differentiation in *Drosophila* [16]. The *Bmotu* expression level was up-regulated when *Bmovo-1* was overexpressed in silkworm ovaries and down-regulated when *Bmovo* was silenced [14], suggesting that *Bmotu* is a target gene, but to date it is still unknown whether BmOVOs can directly bind to the *Bmotu* promoter to regulate its expression. Therefore, to elucidate the molecular mechanism of *Bmovo* gene splicing in regulating the expression of *otu* gene can help us to further understand the regulatory mechanisms of BmOVO on *Bmotu* promoter activity and ovarian development in the silkworm, thus providing the theoretical basis and molecular target for regulating ovarian development and increasing silkworm silk production through genetic manipulation.

Moreover, in *Drosophila*, the small peptide Tal can induce selective hydrolysis of the N-terminal transcriptional repression domain of Ovo/Svb, which leads to a change from transcriptional repressor to a transcriptional activator [17, 18]. A *tal-like* gene (NM_0010998471.1) was also found in *B. mori*, but whether BmOVOs are hydrolyzed by the small peptide encoded by this *tal-like* gene is unknown. In this research, the regulation of the four BmOVO isoforms on *Bmotu* promoter activity was investigated with luciferase reporter assays, the binding site of BmOVO to the *Bmotu* promoter was identified with an electrophoretic mobility shift assay (EMSA) and the BmOVO-1 degradation triggered by the Tal-like small peptide was studied. We found that the BmOVOs are transcriptional activators that directly bind to the *Bmotu* promoter, and degradation of BmOVO-1 at its N-terminus is mediated by the Tal-like small peptide. These results will help us to further understand the regulatory mechanisms of BmOVO on *Bmotu* promoter activity and ovarian development in the silkworm.

## Results

### The four alternative splicing isoforms of BmOVO are transcriptional activators

**Fig. 1:**
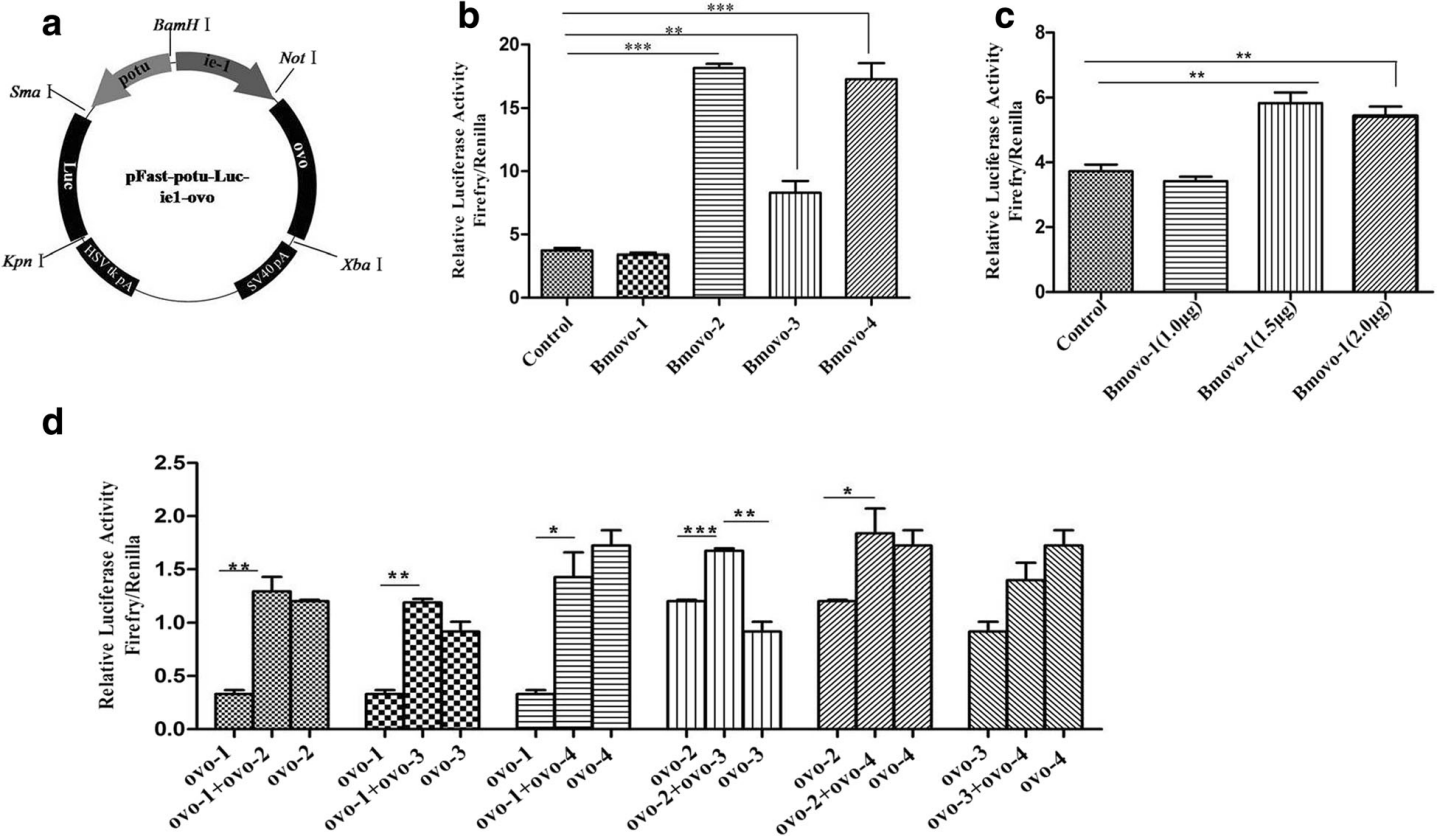
Transcriptional regulation of four BmOVO isoforms on *Bmotu* promoter. **a** Schematic of the recombinant plasmid. The basic vector is pFastBac™Dual vector. Potu represents the *B. mori otu* promoter; ie-1 represents *B. mori* nucleopolyhedrovirus immediate early-1 promoter; ovo represents four Bmovo alternative splicing isoforms of *Bmovo* gene; luc represents *Luciferase* gene; SV40pA represents Simian virus 40 (SV40) polyA signaling sequence; HSVtkpA represents the polyA signaling sequence of herpes simplex virus (HSV) thymidine kinase (TK) gene. Promoters ie1 and potu are responsible for transcription of gene *Bmovo* and *luc* respectively. **b** Luciferase activities in the co-transfected BmN cells with pFast-potu5-Luc-ie1-Bmovo and pRL-TK plasmids. The mixture of pFast-potu5-Luc-ie1-Bmovo (1 × 10^11^ copies) and pRL-TK (1 × 10^10^ copies) plasmids were transfected into BmN cells (10^5^), and the co-transfected BmN cells with pFast-potu5-Luc (1 × 10^11^ copies) and pRL-TK (1 × 10^10^ copies) plasmids was used as a control. **c** 1.0, 1.5 and 2.0 μg of pFast-potu5Luc-ie1-Bmovo1 plasmid was co-transfected into BmN cells (10^5^) respectively with 0.1, 0.15, and 0.2 μg of plasmid pRL-TK, and the co-transfected BmN cells with pFast-potu5-Luc(1.0 μg) and pRL-TK(0.1 μg) plasmids was used as a control. **d** Different combinations of pFast-potu5-Luc-ie1-Bmovo (1 × 10^11^ copies) were co-transfected into BmN cells (10^5^) with pRL-TK (1 × 10^10^ copies). In various combinations, the copies of each pFast-potu5-Luc-ie1-Bmovo plasmid was 5 × 10^10^, and the total copies of plasmids was still 10^11^. Luciferase activities in the cells were determined at 60 h post-transfection, 100 μg protein from the lysed cells was used for luciferase assay (*p < 0.05, **p < 0.01, ***p < 0.001)

To investigative the transcriptional activation or repression of BmOVO isoforms, four luciferase expression vectors (pFast-potu5-Luc-ie1-Bmovo) were constructed. In these vectors, the four spliced isoforms of the *Bmovo* gene (*Bmovo-1*, *− 2*, *− 3* and *− 4*) were controlled by the *B. mori* baculovirus *ie-1* promoter, while *luc* gene expression was driven by the promoter of *Bmotu* (Fig. 1a). A dual-luciferase reporter assay was performed with co-transfection of both pFast-potu5-Luc-ie1-Bmovo (1 × 10^11^ copies) and the reference plasmid pRL-TK (1 × 10^10^ copies) into BmN cells (10^5^ cells). The results showed that BmOVO-2, BmOVO-3 and BmOVO-4 positively regulated *Bmotu* promoter activity, with BmOVO-2 having the highest transcriptional activation activity (Fig. 1b). When low doses of pFast-potu5-Luc-ie1-Bmovo1 were transfected into BmN cells, the transcriptional activation of BmOVO-1 was not significant, but with increasing doses, *Bmotu* promoter activity was enhanced (Fig. 1c). Additionally, the different combinations of luciferase gene expression vectors were co-transfected into BmN cells (Additional file 1: Table S1), and the *Bmotu* promoter activity was detected by dual-luciferase reporter assays. The results showed that when pFast-potu5-Luc-ie1-Bmovo1 was co-transfected with pFast-potu5-Luc-ie1-Bmovo2, pFast-potu5-Luc-ie1-Bmovo3 and pFast-potu5-Luc-ie1-Bmovo4, respectively, *Bmotu* promoter activity was significantly increased compared with transfection with pFast-potu5-Luc-ie1-Bmovo1 alone, while the activity was similar to that for transfection with pFast-potu5-Luc-ie1-Bmovo2, pFast-potu5-Luc-ie1-Bmovo3 or pFast-potu5-Luc-ie1-Bmovo4 alone. These results indicate that the transcriptional activity of the *Bmotu* promoter could be improved by the interaction of BmOVO-1 with BmOVO-2, BmOVO-3 and BmOVO-4, respectively. When pFast-potu5-Luc-ie1-Bmovo2 was co-transfected with pFast-potu5-uc-ie1-Bmovo3, *Bmotu* promoter activity was significantly increased compared with transfection with either pFast-potu5-Luc-ie1-Bmovo2 or pFast-potu5-Luc-ie1-Bmovo3, suggesting that the transcriptional activation of BmOVO-2 and BmOVO-3 was increased by their interaction. The transcriptional activity of the *Bmotu* promoter in the co-transfected BmN cells with both pFast-potu5-Luc-ie1-Bmovo2 and pFast-potu5-Luc-ie1-Bmovo4 was higher than that in the transfected BmN cells with pFast-potu5-Luc-ie1-Bmovo2. This result indicates that the transcriptional activation of BmOVO-2 could be improved by interaction with BmOVO-4. Meanwhile, when pFast-potu5-Luc-ie1-Bmovo3 was co-transfected with pFast-potu5-Luc-ie1-Bmovo4, *Bmotu* promoter activity was not increased compared with transfection with either pFast-potu5-Luc-ie1-Bmovo3 or pFast-potu5-Luc-ie1-Bmovo4, indicating that transcriptional activation of BmOVO-3 was not increased by the interaction with BmOVO-4, *and* vice versa (Fig. 1d).

### A1 and A3 acidic domains of BmOVO-1 are transcriptional repression domains, while A4 and A5 acidic domains are transcription activation domains

BmOVOs are transcription factors with regulatory functions that depend on the N-terminal functional domains. Our previous study indicated that there are six acidic domains (A1–A6) and two basic domains (B1 and B2) at the N-terminus of BmOVO-1, two acidic regions (A4 and A5) and two basic domains (B1 and B2) in BmOVO-2 and only one acidic region (A6) in BmOVO-3 while no known functional domains were detected in BmOVO-4. Furthermore, BmOVO-4 has only one zinc-finger (Z4), while BmOVO-1, − 2 and − 3 have four zinc-fingers (Z1–Z4) at their C-termini (Additional file 1: Figure S1) [14].

**Fig. 2:**
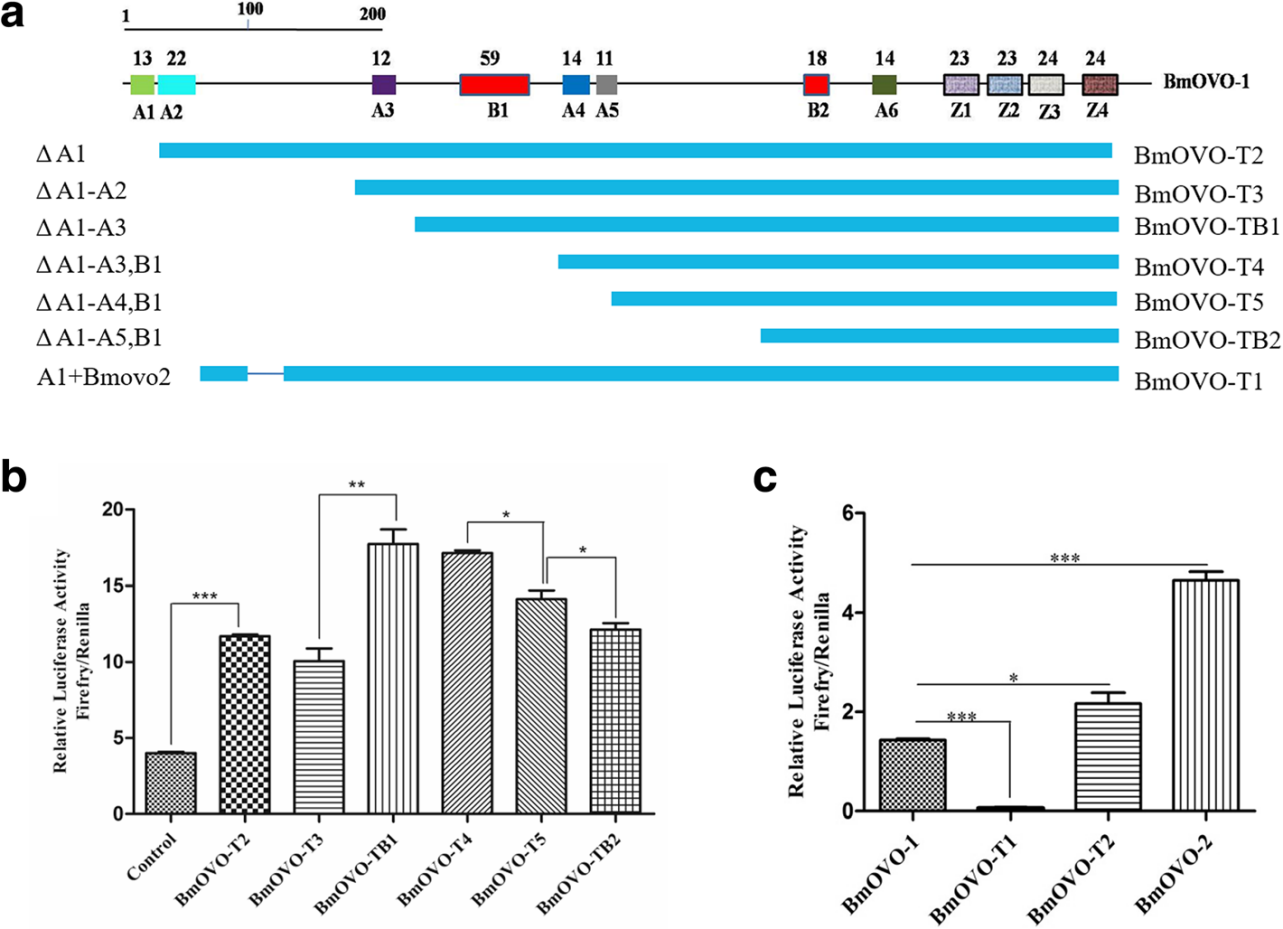
Transcriptional regulation of different domains of BmOVO-1 at the N-terminus on Bm*otu* promoter. **a** Schematic of truncated BmOVO-1. Upper, the domains of BmOVO-1, A represents acidic domain, B represents alkaline domain, Z represents zinc-finger domain, Number on domain means number of amino acid residues in a domain; Lower, the truncated BmOVO-1. BmOVO-T2, A1 region of BmOVO-1 was deleted; BmOVO-T3, A1-A2 region of BmOVO-1 was deleted; BmOVO-TB1, A1-A3 region of BmOVO-1 was deleted; BmOVO-T4, A1-B1 region of BmOVO-1 was deleted; BmOVO-T5, A1-A4 region of BmOVO-1 was deleted; BmOVO-TB2, A1-A5 region of BmOVO-1 was deleted; BmOVO-T1, BmOVO-2 was fused with A1 domain. **b, c** Plasmids with different truncated Bmovo-1(1 × 10^11^ copies) and BmOVO-T1(1 × 10^11^ copies) were respectively cotransfected into BmN cells (10^5^) with plasmid pRL-TK (1 × 10^10^ copies). The co-transfected BmN cells with pFast-potu5-Luc-ie1-Bmovo1(1 × 10^11^ copies) and pRL-TK (1 × 10^10^ copies) plasmids was used as a control. Luciferase activities in the cells were determined at 60 h post-transfection, 100 μg protein from the lysed cells was used for luciferase assay. *p < 0.05, **p < 0.01, ***p < 0.001

To investigate the function of domains which are located at the N-terminus of the protein BmOVO-1, luciferase expression vectors with different truncated *Bmovo-1* expression cassettes (1 × 10^11^ copies) (Fig. 2a) were co-transfected into BmN cells with the reference plasmid pRL-TK (1 × 10^10^ copies). The results showed that both the A1 and A3 domains had transcriptional inhibition on the *Bmotu* promoter and the both A2 and B1 domains didn’t have significant transcriptional regulation, while both A4 and A5 domains had transcriptional activation activities (Fig. 2b). Moreover, when the A1 domain was fused with the N-terminus of BmOVO-2, the transcription activation of the fusion protein decreased sharply compared with BmOVO-2 (Fig. 2c), strongly indicating that the A1 domain functions as a transcriptional repressor.

### Transcriptional regulatory activity of BmOVO is regulated by a small peptide encoded by a *tal-like* gene

**Fig. 3:**
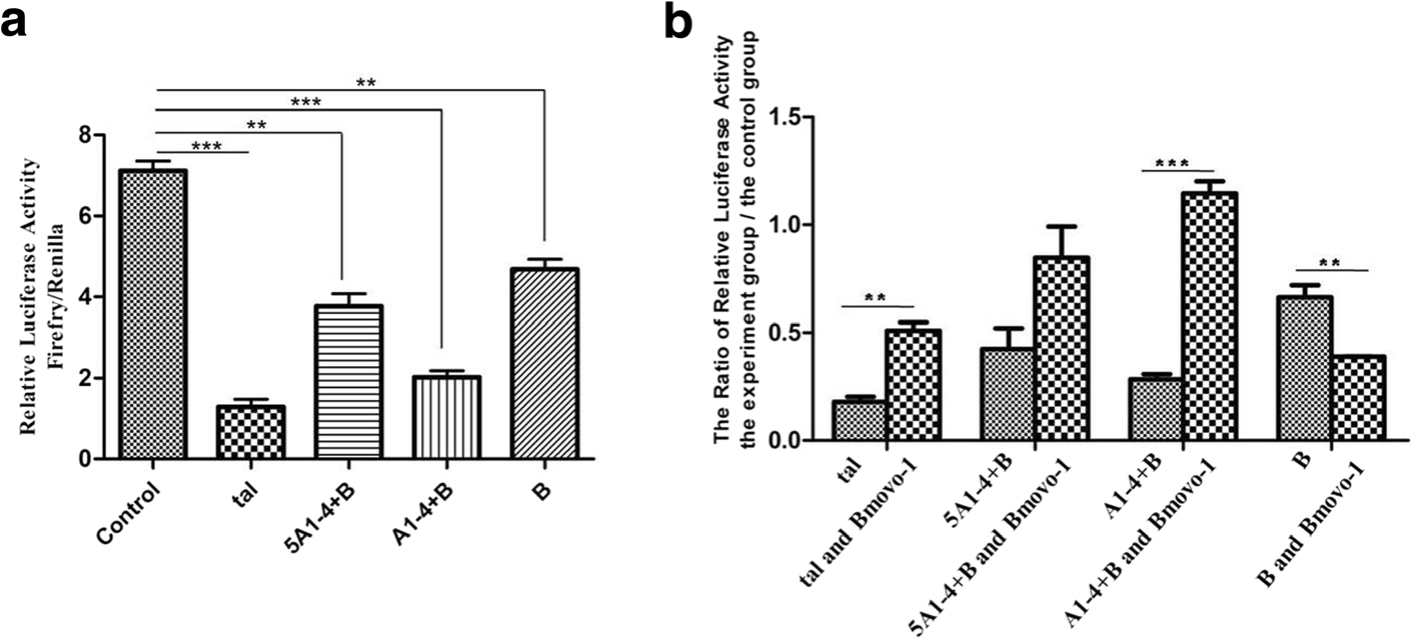
Transcriptional regulatory activity of BmOVO could be regulated by a small peptide encoded by *tal-like* gene. **a** Luciferase activities in the co-transfected BmN cells (10^5^) with plasmids pFast-potu5-Luc (1 × 10^11^ copies), pRL-TK (1 × 10^10^ copies) and *tal-like* expression vectors (1 × 10^11^ copies) were determined at 60 h post-transfection, 100 μg protein from the lysed cells was used for luciferase assay. The co-transfected BmN cells with pFast-potu5-Luc (1 × 10^11^ copies) and pRL-TK (1 × 10^10^ copies) plasmids was used as a control. **b** Plasmids pFast-potu5-Luc-ie1-Bmovo1(1 × 10^11^ copies), pRL-TK (1 × 10^10^ copies) and *tal-like* expression vectors (1 × 10^11^ copies) were co-transfected into BmN cells (10^5^), and the co-transfected BmN cells with pFast-potu5 (1 × 10^11^ copies), pRL-TK (1 × 10^10^ copies) and *tal-like* expression vectors (1 × 10^11^ copies) plasmids was used as a control. Luciferase activities were determined at 60 h post-transfection, 100 μg protein from the lysed cells was used for luciferase assay. The ratio of relative luciferase activity means relative luciferase activity in the experimental group than that in the control group. Tal, a full-length cDNA sequence of *tal-like*; 5A1–4 + B, a sequence containing 5′-non-coding sequence and it’s downstream 1A - 4A with B; A1–4 + B, a sequence containing A1-A4 with B; B, a sequence containing B and its downstream non-coding sequence. (**p < 0.01, ***p < 0.001)

**Fig. 4:**
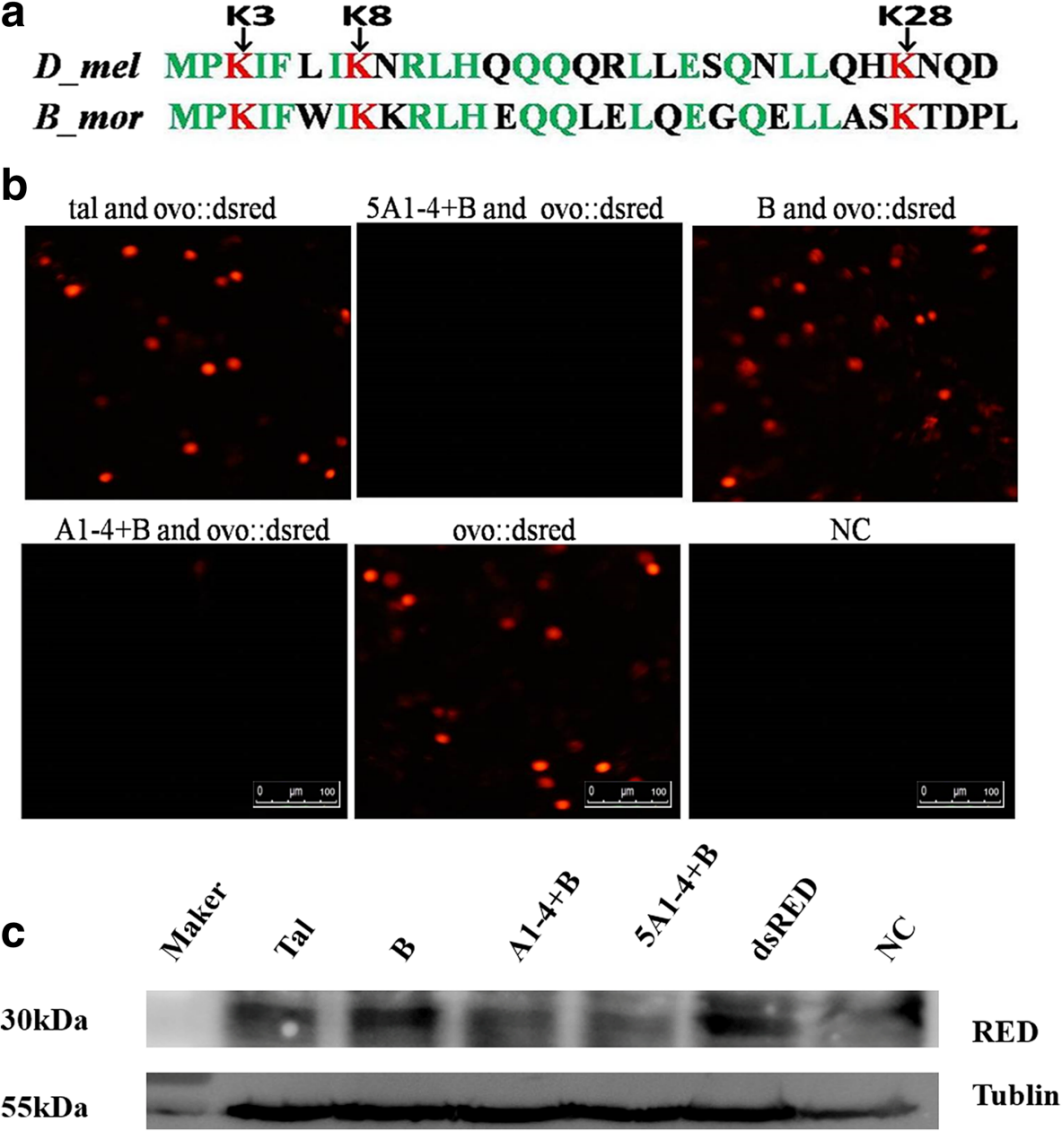
Small peptide can induce the degradation of fusion protein OVO::dsRED. **a** Svb/OVO N-terminal sequences from *Drosophila* (*D_mel*) and *Bombyx mori* (*B_mor*),K3, K8 and K28 respectively represent the third, the eighth and the twenty-eighth Lys residues at Svb/OVO N-terminus (**b**) Observation of red fluorescent cells. Tal and ovo::dsred, the cotransfected BmN cells with a full length of *tal-like* cDNA gene expression vector and DsRed fused with N- terminus 28 residues of BmOVO-1 expression vector. 5A1–4 + B and ovo::dsred, the cotransfected BmN cells with a sequence containing 5′-non coding sequence and it’s downstream 1A - 4A with B of *tal-like* cDNA gene expression vector and DsRed fused with N- terminus 28 residues of BmOVO-1 expression vector. A1–4 + B and ovo::dsred, the cotransfected BmN cells with a sequence containing A1-A4 with B of *tal-like* cDNA gene expression vector and DsRed fused with N- terminus 28 residues of BmOVO-1 expression vector. B and ovo::dsred, the cotransfected BmN cells with a sequence containing B and its downstream non coding sequence of *tal-like* cDNA gene expression vector and DsRed fused with N-terminus 28 residues of BmOVO-1 expression vector. Ovo::dsred, the transfected BmN cells with DsRed fused with N- terminus 28 residues of BmOVO-1 expression vector. NC means natural control representing BmN cells that do not transfect anything. (**c**) Western blotting of cells transfected with tal and ovo::dsred, B and ovo::dsred, 5A1–4 + B and ovo:: dsred, A1–4 + B and ovo::dsred, ovo::dsred. For detection of DsRed, the mouse antibody against RFP was used as a primary antibody, antibody goat anti-mouse HRP used as a secondary antibody. The tubulin was used as internal reference. 30 μg protein from the lysed cells was used for western blotting

Whether the transcriptional regulation of BmOVOs can be regulated by a Tal-like protein in *B. mori* is unclear. In this study, the *tal-like* expression vectors (pIZT/V5-His-tal, pIZT/V5-His-5A1–4 + B, pIZT/V5-His-A1–4 + B and pIZT/V5-His-B) were utilized. The mixture of the *tal-like* expression vector (1 × 10^11^ copies) and pFast-potu5-Luc (1 × 10^11^ copies) was co-transfected into BmN cells with pRL-TK (1 × 10^10^ copies), and the dual-luciferase reporter assay results showed that the *Bmotu* promoter activity was significantly reduced in the transfected cells with *tal-like* expression plasmids compared with the controls, in which the transfected cells did not have *tal-like* expression plasmids, suggesting that the *Bmotu* promoter activity was down-regulated by the *B. mori* Tal-like peptide (Fig. 3a). The *Bmotu* promoter activity in the co-transfected BmN cells with a mixture of the *tal-like* gene expression vector (pIZT/V5-His-tal, pIZT/V5-His-5A1–4 + B or pIZT/V5-His-A1–4 + B) (1 × 10^11^ copies) and pFast-potu5-Luc-ie1-Bmovo1 (1 × 10^11^ copies) was higher than that in the transfected BmN cells with the *tal-like* gene expression vector (1 × 10^11^ copies) alone, but the *Bmotu* promoter activity in the co-transfected BmN cells with a mixture of pIZT/V5-His-B (1 × 10^11^ copies) and pFast-potu5-Luc-ie1-Bmovo1 (1 × 10^11^ copies) was lower than that in the transfected BmN cells with pIZT/V5-His-B (1 × 10^11^ copies), suggesting that *Bmotu* promoter activity was negatively regulated by the Tal-like peptide (Tal, 5A1–4 + B and A1–4 + B) but could be rescued by BmOVO-1 to a certain extent (Fig. 3b). Furthermore, the fusion expression vector pIZT/V5-His-ovodsred containing the fused sequence of the N-terminus (28aa) of BmOVO-1 (Fig. 4a) and the red fluorescent protein (*dsred*) gene was co-transfected into BmN cells with each *tal-like* gene expression vector, respectively (pIZT/V5-His-tal, pIZT/V5-His-5A1–4 + B, pIZT/V5-His-A1–4 + B or pIZT/V5-His-B). The cells containing pIZT/V5-His-tal or pIZT/V5-His-B produced red fluorescence, but that co-transfected with pIZT/V5-His-5A1–4 + B or pIZT/V5-His-A1–4 + B did not (Fig. 4b). The results showed that a signaling band representing DsRed could be clearly found in the co-transfection with pIZT/V5-His-tal or pIZT/V5-His-B, but not in the co-transfection with pIZT/V5-His-5A1–4 + B or pIZT/V5-His-A1–4 + B (Fig. 4c). These results indicate that DsRed fused with 28 aa of the N-terminus of BmOVO-1 were degraded by triggering with 5A1–4 + B or A1–4 + B.

### Transcriptional regulatory activity of BmOVO was regulated by *Dpp*, *Daw*, *Ror2*, *STAT* and *BBx-B8*

Previous studies in our laboratory have found that the genes (*Dpp*, *Daw*, *BBX-B8*, *Ror2*, *STAT*) are involved in important signaling pathway and associated with silkworm growth and development processes [19–21]. To further investigate the transcriptional regulatory function of BmOVO on the *Bmotu* promoter, pFast-potu-Luc-ie1-Bmovo2 (1 × 10^11^ copies) was respectively co-transfected with the pIZT/V5-His, pIZT/V5-His-STAT, pIZT/V5-His-DPP, pIZT/V5-His-Daw, pIZT/V5-His-Ror2 and pIZT/V5-His-BBX-B8 plasmids (1 × 10^11^ copies). The dual-luciferase reporter assay showed that *Bmotu* promoter activity clearly decreased in the cells that were co-transfected with pIZT/V5-His-STAT, pIZT/V5-His-DPP, pIZT/V5-His-Daw, pIZT/V5-His-Ror2 or pIZT/V5-His-BBX-B8 compared with the activity of the cells co-transfected with pIZT/V5-His (Additional file 1: Figure S2), suggesting that *Dpp*, *Daw*, *Ror2*, *STAT* and *BBx-B8* had antagonistic effects on the transcriptional activation of BmOVO-2. These results provide a clue towards understanding the precise regulation of the target gene expression of BmOVO.

### BmOVO binds to the promoter region of *Bmotu*

**Fig. 5:**
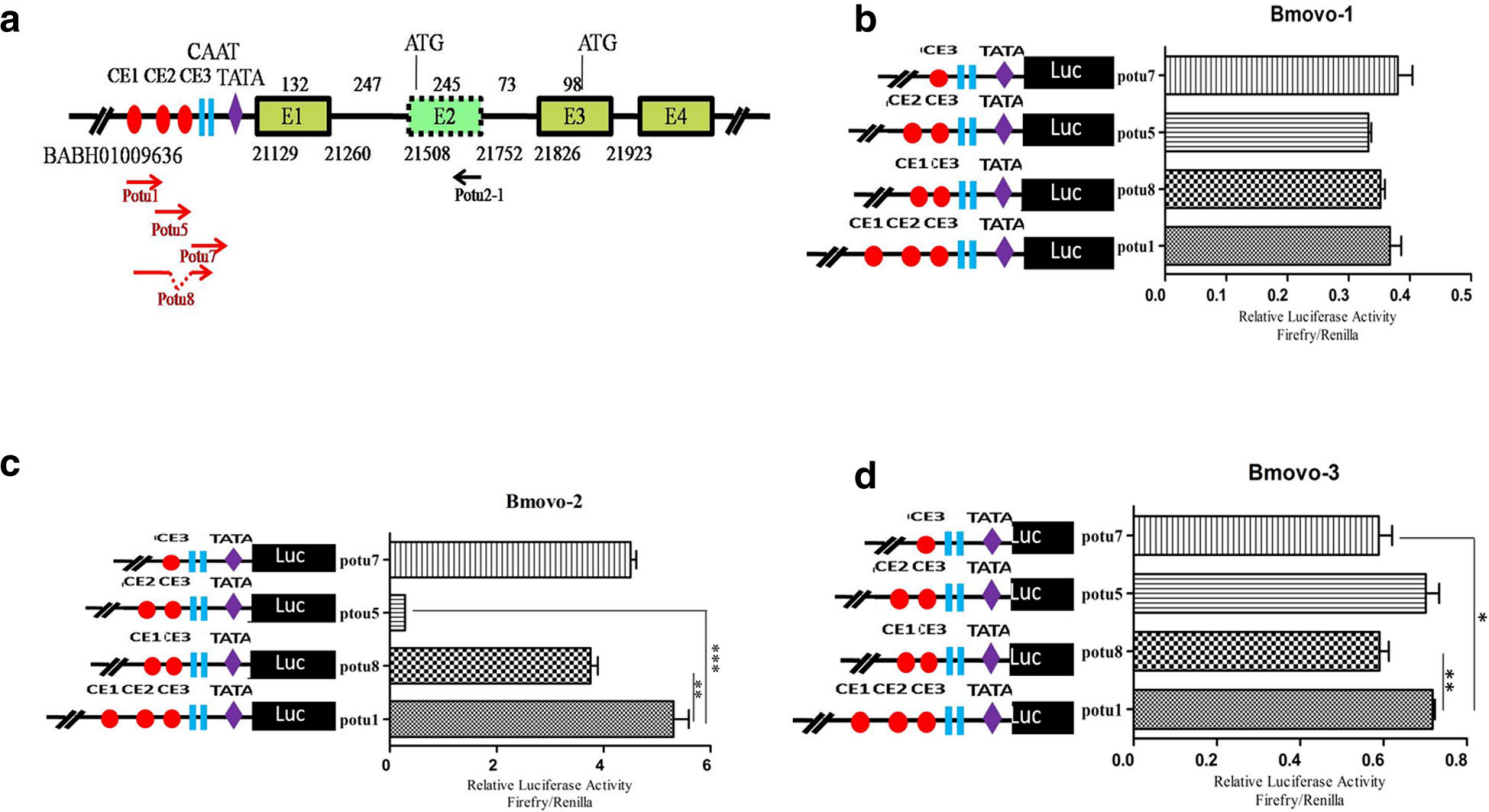
Luciferase activity of different truncated Bm*otu* promoter regulated by BmOVO. **a** Prediction of cis-acting elements on the promoter of Bm*otu* (BABH01009636). BmOVO control element (CE) shows as ●, TATA box as ◆, CAAT box as ▍, primer as →, exon as E, the numbers above the line indicates the size of exon or intron, the number below the line indicate the location of exon in genomic sequence (BABH01009636). **b** Luciferase activity analysis for different truncated Bm*otu* promoter regulated by BmOVO-1. **c** Luciferase activity analysis for different truncated Bm*otu* promoter regulated by BmOVO-2. **d** Luciferase activity analysis for different truncated Bm*otu* promoter regulated by BmOVO − 3. BmN cells (10^5^) were transfected with plasmids containing Bmovo expression cassette driven by different truncated *otu* promoter (1 × 10^11^ copies) and pRL-TK (1 × 10^10^ copies). Luciferase activities in the cells were determined at 60 h post-transfection, 100 μg protein from the lysed cells was used for luciferase assay(*p < 0.05,**p < 0.01, ***p < 0.001)

**Fig. 6:**
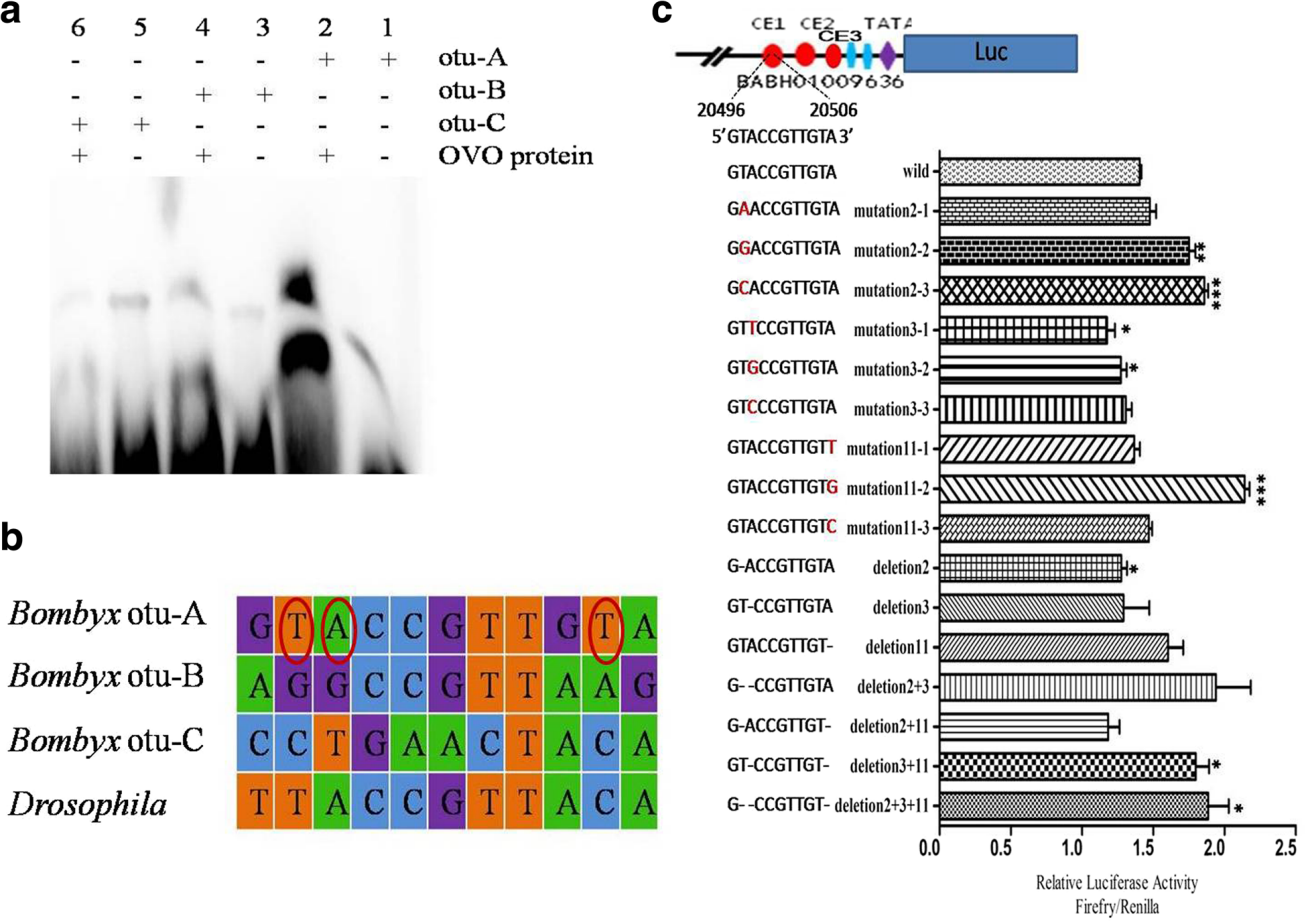
Analysis of the binding elements of BmOVO zinc-finger domain on Bmotu promoter. **a** Binding of BmOVO zinc-finger domain to probes otu-A, otu-B and otu-C. **b** Sequence alignment of the predicted target binding sites of BmOVO protein. **c** BmN cells (10^5^) were transfected with plasmids containing a *luc* gene driven by Bm*otu* promoters with different mutated CE1 element (1 × 10^11^ copies) and pRL-TK (1 × 10^10^ copies). Luciferase activities in the cells were determined at 60 h post-transfection, 100 μg protein from the lysed cells was used for luciferase assay. Mutant bases are shown in red. The deleted base are shown in “-”(*p < 0.05,**p < 0.01,***p < 0.001)

To identify the target binding sites of BmOVO on the *Bmotu* promoter, the potential binding sites of BmOVO on the *Bmotu* promoter (BABH01009636) were predicted by JASPAR CORE (http://jaspar.genereg.net/cgi-bin/jaspar_db.pl) based on the conserved sequence (TTACMGTTACA, where M = A/C) of the *Drosophila* OVO target site. Three candidate binding sites, CE1 (GTACCGTTGTA. nt, 20,496–20,506), CE2 (AGGCCGTTAAG. nt, 20,598–20,608) and CE3 (CCTGAACTACA. nt, 20,728–20,738) were found in the *Bmotu* promoter sequence (Fig. 5a). The activity of different truncated *Bmotu* promoters was investigated with dual-luciferase reporter assays, and the results showed that deletion of the CE1 and CE2 elements didn’t have a significant effect on the *Bmotu* promoter activity regulated by BmOVO-1 (Fig. 5b). When CE1 was deleted, *Bmotu* promoter activity mediated by BmOVO-2 plummeted, while *Bmotu* promoter activity did not change significantly with the deletion of both CE1 and CE2 (Fig. 5c); when CE2 was deleted, *Bmotu* promoter activity regulated by BmOVO-3 decreased, but a change in *Bmotu* promoter activity was not significant when CE1 was deleted (Fig. 5d). The results of EMSA showed that the C-terminal recombinant protein of BmOVO-2 (400–577 aa) bound to the *Bmotu* promoter at the CE1 site (probe otu-A) (Fig. 6a), suggesting that BmOVO directly regulates *Bmotu* gene expression by binding with its promoter. Furthermore, EMSA was carried out with the extracted nucleoproteins from BmN cells, and the results showed that the probes otu-A, otu-B and otu-C could also bind with nucleoproteins (Additional file 1: Figure S3a and b), suggesting that *Bmotu* gene expression could be regulated by other transcription factors. EMSA was carried out with the extracted nucleoproteins and the mutated probe otu-A (otuA-muts), and we found that the second base T mutated to C (T^2^- > C; otuA-mut1) at probe otu-A, causing its ability to bind with the nucleoprotein to be weakened. Additionally, the eleventh base A mutated to G (A^11^- > G; otuA-mut3), leading to enhanced binding capacity, while a clear change was not found when the third base A was mutated to G (A^3^- > G; otuA-mut2) (Additional file 1: Figure S3c). These results indicate that *Bmotu* gene expression is not only regulated by *cis*-regulatory elements, but also by the BmOVO transcription factors and other *trans*-acting regulatory factors. Sequence alignment of the predicted target binding sites (CE1, CE2 and CE3) also showed that three bases (T^2^, A^3^ and A^11^) of the CE1 element might play an important role in BmOVO zinc finger binding to CE1 (Fig. 6b). To confirm this prediction, a dual-luciferase reporter assay was further performed with serial luciferase reporter vectors in which the *luc* gene was controlled by *Bmotu* promoters with different mutated CE1 elements. The results showed that when T^2^ was mutated to G or C, *Bmotu* promoter activity regulated by BmOVO-2 was enhanced. However, when T^2^ was mutated to A, a significant change in *Bmotu* promoter activity was not found. When A^3^ was mutated to T, G or C, the *Bmotu* promoter activity was weakened, while when A^11^ was mutated to G, the *otu* promoter activity increased. Additionally, when T^2^, A^3^ and A^11^ were respectively deleted, there was no significant change in *Bmotu* promoter activity; interestingly, *Bmotu* promoter activity was enhanced with the deletion of all of these bases (T^2^, A^3^ and A^11^) (Fig. 6c). These results indicate that mutation of the CE1 element leads to a change in the advanced structure of the *Bmotu* promoter, which alters its ability to bind to BmOVO and results in a change in the *Bmotu* gene expression level.

## Discussion

Previous studies have shown that the expression profiles of the *Bmovo* genes (*Bmovo-1*, *Bmovo-2*, *Bmovo-3* and *Bmovo-4*) vary in the gonads of the silkworm at different developmental stages [15]. *Bmotu* is a target gene of the BmOVO proteins [15] that is essential for female germline differentiation, suggesting that *Bmotu* gene expression in the ovary of the silkworm at different developmental stages is regulated by different BmOVO isoforms. In the present study, we found that the transcriptional regulatory activity of the four isoforms of BmOVO on *Bmotu* was different: BmOVO-2, BmOVO-3 and BmOVO-4 are transcriptional activators. However, the positive regulatory effects of BmOVO-1 were not significant in cells transfected with a low dose of the Bmovo-1 expression vector, while the positive regulatory effect was increased in the cells transfected with a high dose of this vector, suggesting that BmOVO-1 is also a transcriptional activator. However, in vitro system cannot fully represent the situation in vivo, only to regulating the expression of endogenous BmOVO can verify the regulatory roles of BmOVO isoforms, which needs to be studied at the individual level. It may be necessary to establish BmOVO transgenic silkworm to solve this problem in the future. In *Drosophila*, the *ovo* locus encodes three isoforms, Shaven baby (Svb), OVO-A and OVO-B [7]. OVO-B positively regulates *otu* gene expression, while OVO-A is a repressor [8, 12]. However, we did not find which of the known BmOVO isoforms a transcriptional repressor was. We inferred that the *Bmovo* locus might also encode a novel BmOVO based on N-terminal domain analysis of the known BmOVO, which may be a transcriptional repressor. Our previous study predicted that BmOVO-2 may be a transcriptional activator [14], because the N-terminal domain of BmOVO-2 contains a serine-rich region (57%), an acidic domain (pI = 3.57) and a glycine-rich region, which also exists in the transcriptional activator OVO-B (56%) [4]. Our results showed that BmOVO-2 significantly increased the activity of the *Bmotu* promoter, indicating that BmOVO-2 has a positive regulatory effect, which was consistent with the predicted results. BmOVO-3 significantly increased the activity of the *Bmotu* promoter. The N-terminus of the BmOVO-3 protein has only one acidic domain (A6), so it can be considered that the A6 acidic domain of BmOVO-3 is a transcriptional activation domain. BmOVO belongs to the C_2_H_2_-type zinc finger family, and generally three or more of these zinc fingers are required for binding to target DNA. BmOVO-4 only has one zinc finger structure and no known regulatory domain at its N-terminus, but BmOVO-4 positively regulated *Bmotu* promoter activity; therefore, we hypothesized that BmOVO-4 may interact with other *trans*-acting factors to lead to the increased expression of target genes. Usually, transcription factors bind to target DNA sequences in homodimers or heterodimers. It was found that the transcriptional activity of the *Bmotu* promoter could be improved by the interaction of BmOVO-1 with BmOVO-2, BmOVO-3 and BmOVO-4, the interaction of BmOVO-2 with BmOVO-3 and the interaction of BmOVO-2 with BmOVO-4, suggesting that these four BmOVO isoforms compete for binding sites and cross-regulate the expression of target genes as homodimers and heterodimers. Our previous study showed that BmOVO-1, BmOVO-2 and BmOVO-4 are mainly expressed in the ovaries, while BmOVO-3 is mainly expressed in the testes [14]; therefore, BmOVO-3 may perform transcriptional regulation as a homodimer.

OVO-B and OVO-A are transcription factors with opposing regulatory activities due to different effector domains at their N-termini. In *Drosophila* OVOs, one glycine-rich region, two acidic regions and an extensive glutamine/histidine-rich region comprise the activation region; a charged basic region and a serine-rich domain comprise the repression region [13]. The distribution and number of acidic and basic domains at the N-termini of BmOVO sequences differed from *Drosophila* OVO, suggesting differences in function between BmOVO and *Drosophila* OVO. In this study, we found that the A1 and A3 acidic domains at the N-terminus of BmOVO-1 were transcriptional repression regions, and the A4 and A5 acidic domains were transcriptional activation regions, while the transcriptional regulation of the A2 and B1 domains was not significant. The four BmOVO isoforms have different effector domains at their N-termini, which result in differences in transcriptional regulation activities between the BmOVO isoforms; this result was confirmed by dual-luciferase reporter assays. It has been found that when *Drosophila* OVO-B was fused with the transcriptional repression domain of *Drosophila* OVO-A, *Drosophila* OVO-B was changed from a transcriptional activator into a transcriptional repressor [13]. A similar phenomenon was found in BmN cells, where the A1 acidic domain of BmOVO-1 also turned BmOVO-2 from a transcriptional activator into a transcriptional repressor. This result strongly suggests that A1 is a transcriptional repression domain. Domains containing a high proportion of acidic amino acids generally play a transcriptional activation role [19, 22, 23], but our study indicated that the A1 and A3 acidic domains in BmOVO-1 did not. Thus, we conjectured that the N-terminal domain of BmOVO-1 changes its transcriptional regulatory activity through its interaction with other proteins and/or a change of the binding capacity of BmOVO-1 to *cis*-acting factors.

In *Drosophila*, the small peptide Tal induces selective hydrolysis of the N-terminal transcriptional repression domain of Ovo/Svb mediated by the proteasome, which leads to a switch of Ovo/Svb from a transcriptional repressor to a transcriptional activator [17, 18]. The 31 residues of Ovo/Svb at its N-terminus act as a Tal-dependent degradation signal, while three lysines (K3, K8, K23) within this region play a key role in Ovo/Svb processing [18]. It was found that these 31 residues were highly conserved between BmOVO-1 and Ovo/Svb and the small peptide Tal-like encoded by the silkworm could trigger the hydrolysis of DsRed fused with the N-terminal 28 residues of BmOVO-1. BmOVO-1 can partially rescue *Bmotu* promoter activity that is repressed by the small peptide Tal-like, suggesting that the transcriptional activation of BmOVO-1 is increased by the hydrolysis of the N-terminal transcriptional repression domain of BmOVO-1, triggered by Tal-like. These results indicate that BmOVO protein levels are not only determined by the transcriptional level of the different isoforms, but also by post-translational degradation.

OVO belongs to a large transcription factor family with conservation of function over evolutionary distance. A previous study found that OVO transcription factors are present in *Drosophila, B. mori,* planarians, nematodes, zebrafish and mouse, and they interact with multiple signaling pathways and regulate growth and development [1, 3, 6]. In this study, we found that both *Dpp* and Daw, members of the TGF-β family, inhibited the transcriptional activation of BmOVO-2 on the *Bmotu* promoter. We speculated that over-expression of *Dpp* and *Daw* activated the TGF-β signaling pathway [20], and then down-regulated the activity of the *Bmotu* promoter together with BmOVO. Additionally, overexpression of BBX-B8, Ror2 and STAT also reduced *Bmotu* promoter activity. The expression of genes related to growth and development could be regulated by over-expression of the insulin-like peptide BBX-B8, an extracellular peptide [21]. Additionally, BmOVO was localized in the nucleus; therefore, the decline in *Bmotu* promoter activity was not caused by the direct interaction of BBX-B8 with BmOVO. Previous studies have shown that the classical Wnt signaling pathway is mediated by Ror2 in human lung cancer cells [24]. *Bmotu* promoter activity regulated by BmOVO may have been down-regulated by activating the Wnt signaling pathway mediated by Ror2 in the silkworm. STAT also regulates gene expression by binding to the promoter of target genes [25], which may hamper the binding of BmOVO to the promoter, and results in the decline of Bmotu promoter activity. These results provided new clues to later research.

There are 3 CE elements (CE1, CE2, CE3) on *Bmotu* promoter predicted by bioinformatics which were the binding site of zinc finger protein. Dual-luciferase reporter assay showed that the activity of *Bmotu* promoters with diverse deletion of CE elements was different for BmOVO-1, BmOVO-2 and BmOVO-3, suggesting that the relationship of epistatic properties of the CE elements in the *Bmotu* promoter and the activity of *Bmotu* promoters is complex. Moreover, we found that BmOVO binds to the CE1 element (GTACCGTTGTA) located on the *Bmotu* promoter, while the nuclear proteins extracted from BmN cells could bind to the *Bmotu* promoter at three sites (CE1, GTACCGTTGTA; CE2, AGGCCGTTAAG; CE3, CCTGAACTACA) by EMSA. These results suggest that *Bmotu* gene expression can be regulated by other transcription factors in addition to BmOVO, but these transcription factors need to be further screened and verified. It was also found that T^2^, A^3^ and A^11^ in the CE1 sequence might play an important role in binding of BmOVO to the *Bmotu* promoter. *Bmotu* promoter activity was altered with the mutation of the CE1 sequence and the deletion of the CE1 and CE2 elements. This may be related to changes in the *Bmotu* promoter structure. A study investigating the crystal structure of the zinc finger protein-DNA complex showed that the zinc finger was inserted into the large groove of the DNA double helix through its α-helix to combine with DNA [26]. Mutations in the CE1 sequence may change the structure of the DNA double helix, affecting binding of BmOVO to the DNA double helix and resulting in a change of *Bmotu* promoter activity. These results are helpful to further understand the regulatory mechanisms of BmOVO on Bmotu promoter activity and ovarian development in the silkworm.

## Conclusion

In conclusion, the *Bmotu* promoter activity was positively regulated by four BmOVO isoforms and the BmOVO isoforms could bind to the *Bmotu* promoter. These results will help us to further understand the regulatory mechanisms of BmOVO on *Bmotu *promoter activity and ovarian development in the silkworm, thus providing the theoretical basis and molecular target for regulating ovarian development and increasing silkworm silk production through genetic manipulation. In addition, we detected the interaction between the key genes of silkworm important pathway and *Bmovo* gene, and hope to provide a new clue for the further study of the regulatory role of BmOVO.

## Methods

### Cell culture and transient transfection

The BmN cell line originating from the silkworm ovary was stored in our lab and cultured at 26 °C in TC-100 medium (AppliChem, Darmstadt, Germany) containing 10% fetal bovine serum (FBS) (Gibco-BRL, Gaithersburg, Maryland, USA).

Cells were seeded at 1 × 10^5^/well in 24-well culture plates. Expression vectors were transfected into the BmN cells using Lips2000 (Roche, Basel, Switzerland). After 4 h incubation in TC-100 medium without FBS, the medium was replaced with new TC-100 medium with 10% (*v*/v) FBS, and the cells were incubated for 60 h.

### Plasmids and vector constructs

The plasmid pRL-TK (Promega, Carlsbad, CA, USA) was kindly provided by Dr. Wei of Soochow University. We also utilized the following plasmids, which were constructed for a previous study, including pIZT/V5-His-STAT containing the signal transduction and transcription activation factor (*STAT*) gene [25], pIZT/V5-His-DPP containing the decapentaplegic (*Dpp)* gene and pIZT/V5-His-Daw containing dawdle (*Daw*), both of which are members of the transforming growth factor-β(TGF-β family) [20], pIZT/V5-His-Ror2 containing the tyrosine kinase-like orphan receptor (*Ror2*) gene (AK385238.1), which is a Wnt pathway regulatory factor, and pIZT/V5-His-BBX-B8 containing the insulin-like peptide *BBx-B8* [21] (Additional file 1: Table S2).

To generate the luciferase expression vector, the polyhedrin promoter and the p10 promoters were removed from the pFastBac™Dual vector by *Sma*I and *Bam*HI digestion. The promoter of *Bmotu*, amplified from silkworm genomic DNA (*Dazao* strain) with the primers Potu5/Potu2–1 (Additional file 1: Table S3), was cloned into the *Sma*I and *Bam*HI sites of a pFastBac™Dual vector (Invitrogen) to generate pFast-potu5. The luciferase (*luc*) gene amplified from pGL3 (Promega, Madison, WI, USA) with the primers Luc1/Luc2 (Additional file 1: Table S3) was inserted into the *Kpn* I and *Sma* I sites of pFast-potu5 to obtain the vector pFast-potu5-Luc, and then the *B. mori* baculovirus *ie-1* promoter amplified from pSK-ie-1 with the primers oie-1/oie-2 was cloned into pFast-potu5-Luc to construct the vector pFast-potu5-Luc-ie1 by *Bam*H I and *Not* I. Finally, *Bmovo-1*, *− 2*, *− 3* and *− 4* were amplified using the primers ovo-A1/ovo-A2, ovo-B1/ovo-A2, ovo-C1/ovo-A2 and ovo-D1/ovo-A2, respectively (Additional file 1: Table S3) from a plasmid containing the *Bmovo* gene [14]. These genes were then cloned into the *Not* I and *Xba*I sites of pFast-potu5-Luc-ie1 to generate the vector pFast-potu5-Luc-ie1-Bmovo, in which the *luc* and *Bmovo* genes are controlled by the *Bmotu* and ie-1 promoters, respectively. To know if BmOVO-1 was affected by dosage levels, 1.0, 1.5 and 2.0 μg of pFast-potu5Luc-ie1-Bmovo1 plasmid was co-transfected into BmN cells (10^5^) respectively with 0.1, 0.15, and 0.2 μg of plasmid pRL-TK, and the co-transfected BmN cells with pFast-potu5-Luc(1.0 μg) and pRL-TK(0.1 μg) plasmids was used as a control.

There are six acidic domains (A1, aa, 13–25. A2, aa, 30–51. A3, aa, 180–191. A4, aa, 339–352. A5, aa, 362–372. A6, aa, 600–613) and two basic domains (B1, aa,253–311. B2, aa,540–557) at the N-terminus of BmOVO-1 [14]. In order to explore the transcriptional regulatory activity of the effector domains of BmOVO, truncated *Bmovo* (GU477588) sequences (*Bmovo*-T2, nt,88–2421. *Bmovo*-T3, nt,538–2421. *Bmovo*-TB1, nt,757–2421. *Bmovo*-T4, nt,1015–2421. *Bmovo*-T5, nt,1084–2421. *Bmovo*-TB2, nt,1618–2421) amplified from *Bmovo-1* [14] with the designed primers ovo-T2/ovo-A2, ovo-T3/ovo-A2, ovo-TB1/ovo-A2, ovo-T4/ovo-A2, ovo-T5/ovo-A2 and ovo-TB2/ovo-A2 (Additional file 1: Table S3) based on the sequence of the *Bmovo-1* gene was cloned into the pFast-potu5-Luc-ie1 to construct the vectors p*Bmovo*-T2, *pBmovo*-T3, p*Bmovo*-TB1, p*Bmovo*-T4, *pBmovo*-T5 and *pBmovo*-TB2, respectively. The A1, A1–A2*,* A1–A3, A1–B1, A1–A4 and A1–A5 regions were deleted in p*Bmovo*-T2, p*Bmovo*-T3, p*Bmovo*-TB1, p*Bmovo*-T4, *pBmovo*-T5 and *pBmovo*-TB2, respectively. To investigate the function of the A1 domain, *Bmovo-2* with the A1 region amplified from the *Bmovo-2* gene with the primers ovo-T1/ ovo-A2 (Additional file 1: Table S3) was cloned into pFast-potu5-Luc-ie1 to generate *Bmovo*-T1, in which the A1 domain was fused to the N-terminus of BmOVO-2.

To explore the functions of *cis*-acting elements located on the *Bmotu* promoter (BABH01009636), different *Bmotu* promoters that varied in length (potu1 with control element (CE) CE1, CE2 and CE3, nt, 20,464–21,524. potu5 with CE2 and CE3, nt, 20,538–21,524. potu7 with CE3, nt, 20,665–21,524. potu8 with CE1 and CE3, nt, 20,665–21,524) were amplified from silkworm genomic DNA with the primer pairs potu1/potu2–1, potu5/ potu2–1, potu7/potu2–1 and potu8/potu2–1, respectively (Additional file 1: Table S3), and then cloned into the vector pFast-Luc-ie1-Bmovo to generate serial luciferase reporter vectors. Furthermore, the promoters with mutations or deletions at the CE1 (GTACCGTTGTA) region were amplified with different primers (Additional file 1: Table S3) and cloned into the vector pFast-Luc-ie1-Bmovo2 to construct serial luciferase reporter vectors in which the *luc* gene was controlled by the *Bmotu* promoters with the mutated CE1 region.

**Fig. 7:**
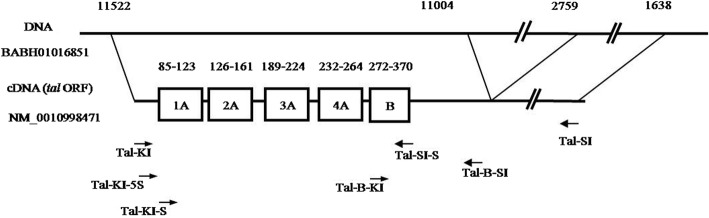
Schematic of *tal-like* cDNA (NM_0010998471) and its coded sORF. The numbers above the figure indicate the location of *tal-like* gene on genomic DNA (BABH01016851), and the numbers in the second row indicate the location of sORF on the *tal-like* cDNA sequence (NM_0010998471). Primers were showed as →

The *tal* gene family is at least 440 million years old and *tal* gene was the most thoroughly studied in *Drosophila*. Its homologs were also found in other species, including *Bombyx mori* [27, 28]. The transcript of the *B. mori tal-like* gene (NM_0010998471) has five small open reading frames (sORFs 1A–4A and B), of which 1A, 2A, 3A, 4A and 4B encode 12, 11, 11, 10 and 32 amino acid residues, respectively (Fig. 7) [29]. In previous study, a full length cDNA sequence of *tal-like* (NM_0010998471.1, nt:1–1616), a sequence containing the 5′-non coding sequence and the downstream sequence 1A–4A with B (5A1–4 + B) (NM_0010998471.1, nt, 9–368), a sequence containing A1–A4 with B (A1–4 + B) (NM_0010998471.1, nt, 80–368) and a sequence containing B and its downstream non coding sequence (B) (NM_0010998471.1, nt, 267–507) were cloned into the insect cell expression vector pIZT-V5/His (Invitrogen) to construct the *tal-like* expression vectors pIZT/V5-His-tal, pIZT/V5-His-5A1–4 + B, pIZT/V5-His-A1–4 + B, and pIZT/V5-His-B, respectively [29]. In this study, these plasmids were used to investigate whether the transcriptional regulation of the BmOVOs is regulated by the Tal-like protein in *B. mori*.

In *Drosophila*, the small peptide Tal triggers the hydrolysis of the N-terminal transcriptional repression domain of Ovo/Svb, which leads to a change from a transcriptional repressor to a transcriptional activator [17, 18]. To investigate whether the degradation of BmOVO-1 is mediated by the small peptide encoded by the *tal-like* gene in the silkworm, the *dsRed* reporter gene fused with the N-terminal domain sequence (28 aa) of BmOVO-1 was amplified with the primer pairs ovo1-dsred-F/ovo1-dsred-R (Additional file 1: Table S3), and the obtained fragment was inserted into the pIZT/V5-His (Invitrogen) to generate the vector pIZT/V5-His-ovodsRED.

### Luciferase reporter assay

Reporter activity was determined using the Dual-Luciferase Reporter Assay System (Promega, Madison, WI, USA) according to the manufacturer’s instructions. Briefly, BmN cells were lysed for 15 min at room temperature using 1× passive lysis buffer which is made from the dilution of the 5 × passive lysis (Promega, Madison, WI, USA), and then the lysed cells were collected and centrifuged at 12,000 g for 10 min. The supernatant was used for the determination of luciferase activity. Luciferase activity was measured using GloMax Multi Jr. at 490 nm (Promega, Madison, WI, USA). T-test was performed for statistical analysis by the software Graphpad Prism5.

### Expression of the BmOVO zinc-finger domain in *Escherichia coli*

The sequence encoding the last 178 aa containing the four zinc-finger domains of BmOVO was amplified with the primer pairs Bmovo2–3/Bmovo2–2 (Additional file 1: Table S3) and was then cloned into the pET28a (+) vector (Invitrogen) for the expression of the recombinant BmOVO zinc-finger domain in *E. coli*. The recombinant protein BmOVO zinc-finger domain with 6-His-tagged was purified with Ni-NTA agarose (Jinyitai, Wuhan, China) according to the manufacturer’s instructions. Then, the refolding of the BmOVO zinc-finger domain was carried out by dialysis in TGN buffer (50 mM Tris-base, 0.5 mM EDTA, 50 mM NaCl, 1% arginine, 10% glycerol, 5 mM GSSG and 2 mM DTT).

### SDS-PAGE and western blotting

30 μg proteins lysed from the transfected cells were subjected to SDS-PAGE, and then transferred to a polyvinylidene fluoride membrane (Roche). The anti-RFP antibody (Abcam, Cambridge, UK) was used, and immunoblotting with anti-β-tubulin antibody (Proteintech, Chicago, IL, USA) was conducted as internal control. The signals were measured using an Enhanced Chemiluminescence (ECL) western blot detection kit (Sangon).

### Electrophoretic mobility shift assay (EMSA)

*Drosophila* OVO directly binds to the sequence TTACMGTTACA, where M = A/C, of the target promoter [8]. To investigate the binding sites of BmOVO, Biotin-labeled DNA probes (otu-A, otu-B and otu-C) (Table 1) and mutated otu-A probes (otuA-mut1, otuA-mut2 and otuA-mut3) (Additional file 1: Table S4) were synthesized (Sangon, Shanghai, China) based on the sequence of the predicted binding sites located on the *Bmotu* promoter using JASPAR CORE (http://jaspar.genereg.net/cgi-bin/jaspar_db.pl). EMSA was performed following the manufacturer’s instructions with the LightShift Chemiluminescent EMSA kit (Thermo, Rockford, Illinois, USA). Briefly, a binding reaction was performed in a final volume of 20 μl, containing 1 μl Poly dI.dC, 1 μl NP-40, 1 μl KCl, 1 μl glycerol, 2 μl 10× binding buffer, 0.75 pM DNA probe and 0.02 mg/ml recombinant BmOVO zinc-finger protein (or 0.175 mg/ml nucleoproteins), followed by incubation for 20 min at room temperature. Then, the recombinant protein binding probe was run on a 6% polyacrylamide gel and transferred to a Hybond-N^+^ membrane (Roche, Basel, Switzerland). The membranes were dried and ultraviolet-crosslinked at 120 mJ/cm^2^ using a UV-light crosslinking instrument. Then, biotin-labeled DNA was detected with the Chemiluminescent Nucleic Acid Detection Module kit (Thermo, USA). Finally, the signal was visualized by phosphorimaging (Clinx, ChemiScope 6300, Shanghai, China).

**Table 1:** Sequence of DNA probe

Probes	Sequences (5′-3′)
otu-A	Biotin-GCCCCTAAAATGTACCGTTGTAACTTCTGT
	Biotin-ACAGAAGTTACAACGGTACATTTTAGGGGC
otu-B	Biotin-GAAAGATAGGCCGTTAAGCGCATCGCACA
	Biotin-TGTGCGATGCGCTTAACGGCCTATCTTTC
otu-C	Biotin-ATGTTATAATTCCTGTAACTACAGACAGGGC
	Biotin-GCCCTGTCTGTAGTTACAGGAATTATAACAT
otuA-mutant1	Biotin-GCCCCTAAAATGCACCGTTGTAACTTCTGT
	Biotin-ACAGAAGTTACAACGGTGCATTTTAGGGGC
otuA-mutant2	Biotin-GCCCCTAAAATGTGCCGTTGTAACTTCTGT
	Biotin-ACAGAAGTTACAACGGCACATTTTAGGGGC
otuA-mutant3	Biotin-GCCCCTAAAATGTACCGTTGTGACTTCTGT
	Biotin-ACAGAAGTCACAACGGTACATTTTAGGGGC

The sequence with underline indicates the predicted binding sites

## Additional file

Additional file 1: **Figure S1.** Structural domain analysis of BmOVOs. A represents acidic domain. B represents alkaline domain. Z represents zinc-finger domain. Number on domain means number of amino acid residues in a domain. **Figure S2.** Transcriptional Regulatory activity of BmOVO could be regulated by *Dpp*, *Daw*, *Ror2*, *STAT* and *BBx-B8*.The mixture of pFast-potu5-Luc-ie1-Bmovo2(1 × 10^11^ copies) and pRL-TK (1 × 10^10^ copies) plasmids was respectively co-transfected with pIZT/V5-His, pIZT/V5-His-STAT, pIZT/V5-His-DPP, pIZT/V5-His-Daw, pIZT/V5-His-Ror2 and pIZT/V5-His-BBX-B8 plasmids (1 × 10^11^ copies) into BmN cells (10^5^), and the co-transfected BmN cells with pFast-potu5-ie1-Bmovo2 (1 × 10^11^ copies) and pRL-TK (1 × 10^10^ copies) plasmids was used as a control. Luciferase activities in the cells were determined at 60 h post-transfection, 100 μg protein from the lysed cells was used for luciferase assay (*p < 0.05, **p < 0.01, ***p < 0.001). **Figure S3.** The Bm*otu* promoter could bind to nucleoproteins. (a) analysis of the binding of probe otu-A to different concentrations of nucleoproteins, lane1–5: the final concentration of nucleoproteins is 0, 0.175, 0.35, 0.525, 0.7 mg/mL; (b) otu-B and otu-C probes binding to nucleoproteins; (**c**) otu-A, otuA-mut1, otuA-mut2 and otuA-mut3 binding to the nucleoproteins. **Table S1.** Different combinations of *luc* expression plasmids. **Table S2.** The plasmids used in this study. **Table S3.** The primers used in this paper. The sequence with underline indicates the enzyme sites, and with wave underline indicates the binding site CE1. Base with frame indicates the mutation. In primer ovo1-dsred-F, the double underline was 5′-terminal sequence of dsred gene, the sequence with boldface were the coding sequence of N- terminus 28 residues of BmOVO-1. **Table S4.** Sequence of mutant DNA probe. Sequence of mutant DNA probe. The sequence with underline indicates the predicted binding sites, and base with frame indicates the mutation. (DOCX 445 kb)

## Abbreviations

*BBx-B8*: The insulin-like peptide BBx-B8
*Bmotu*: *B. mori* ovarian tumor
CE: Control element
*Daw*: Dawdle
*Dpp*: Decapentaplegic
EMSA: Electrophoretic mobility shift assay
FBS: Fetal bovine serum
*Otu*: Ovarian tumor
*Ror2*: The tyrosine kinase-like orphan receptor
sORF: small open reading frames
*STAT*: The signal transduction and transcription activation factor
Z: Zinc-fingers
