# Supplementary material for: Functional characterization of BmOVOs in silkworm, Bombyx mori
Source: BMC Genomics. 2019 May 6;20:342. doi: 10.1186/s12864-019-5697-y (PMC6503385; doi:10.1186/s12864-019-5697-y)
Supplement: Supplementary file 1 — Figure S1. Structural domain analysis of BmOVOs. A represents acidic domain. B represents alkaline domain. Z represents zinc-finger domain. Number on domain means number of amino acid residues in a domain. Figure S2. Transcriptional Regulatory activity of BmOVO could be regulated by Dpp, Daw, Ror2, STAT and BBx-B8.The mixture of pFast-potu5-Luc-ie1-Bmovo2(1 × 10^11^ copies) and pRL-TK (1 × 10^10^ copies) plasmids was respectively co-transfected with pIZT/V5-His, pIZT/V5-His-STAT, pIZT/V5-His-DPP, pIZT/V5-His-Daw, pIZT/V5-His-Ror2 and pIZT/V5-His-BBX-B8 plasmids (1 × 10^11^ copies) into BmN cells (105), and the co-transfected BmN cells with pFast-potu5-ie1-Bmovo2 (1 × 10^11^ copies) and pRL-TK (1 × 10^10^ copies) plasmids was used as a control. Luciferase activities in the cells were determined at 60 h post-transfection, 100 μg protein from the lysed cells was used for luciferase assay (*p < 0.05, **p < 0.01, ***p < 0.001). Figure S3. The Bmotu promoter could bind to nucleoproteins. (a) analysis of the binding of probe otu-A to different concentrations of nucleoproteins, lane1–5: the final concentration of nucleoproteins is 0, 0.175, 0.35, 0.525, 0.7 mg/mL; (b) otu-B and otu-C probes binding to nucleoproteins; (c) otu-A, otuA-mut1, otuA-mut2 and otuA-mut3 binding to the nucleoproteins. Table S1. Different combinations of luc expression plasmids. Table S2. The plasmids used in this study. Table S3. The primers used in this paper. The sequence with underline indicates the enzyme sites, and with wave underline indicates the binding site CE1. Base with frame indicates the mutation. In primer ovo1-dsred-F, the double underline was 5′-terminal sequence of dsred gene, the sequence with boldface were the coding sequence of N- terminus 28 residues of BmOVO-1. Table S4. Sequence of mutant DNA probe. Sequence of mutant DNA probe. The sequence with underline indicates the predicted binding sites, and base with frame indicates the mutation. (DOCX 445 kb) [file 12864_2019_5697_MOESM1_ESM.docx]

# Additional file 1. Supplementary figures and supplementary tables

**
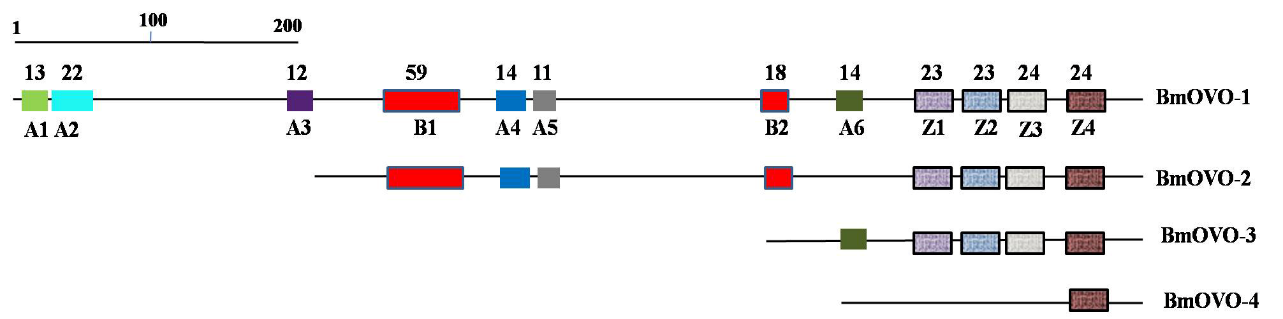
**

Figure S1. Structural domain analysis of BmOVOs.

**

**

Figure S2. Transcriptional Regulatory activity of BmOVO could be regulated by *Dpp*, *Daw*, *Ror2*, *STAT* and *BBx-B8*.The mixture of pFast-potu5-Luc-ie1-Bmovo2(1×10^11^ copies) and pRL-TK (1×10^10^ copies) plasmids was respectively co-transfected with pIZT/V5-His, pIZT/V5-His-STAT, pIZT/V5-His-DPP, pIZT/V5-His-Daw, pIZT/V5-His-Ror2 and pIZT/V5-His-BBX-B8 plasmids (1×10^11^ copies) into BmN cells (10^5^), and the co-transfected BmN cells with pFast-potu5-ie1-Bmovo2 (1×10^11^ copies) and pRL-TK (1×10^10^ copies ) plasmids was used as a control. Luciferase activities in the cells were determined at 60 h post-transfection, 100μg protein from the lysed cells was used for luciferase assay (**p*<0.05, ***p*<0.01, ****p*<0.001).

**
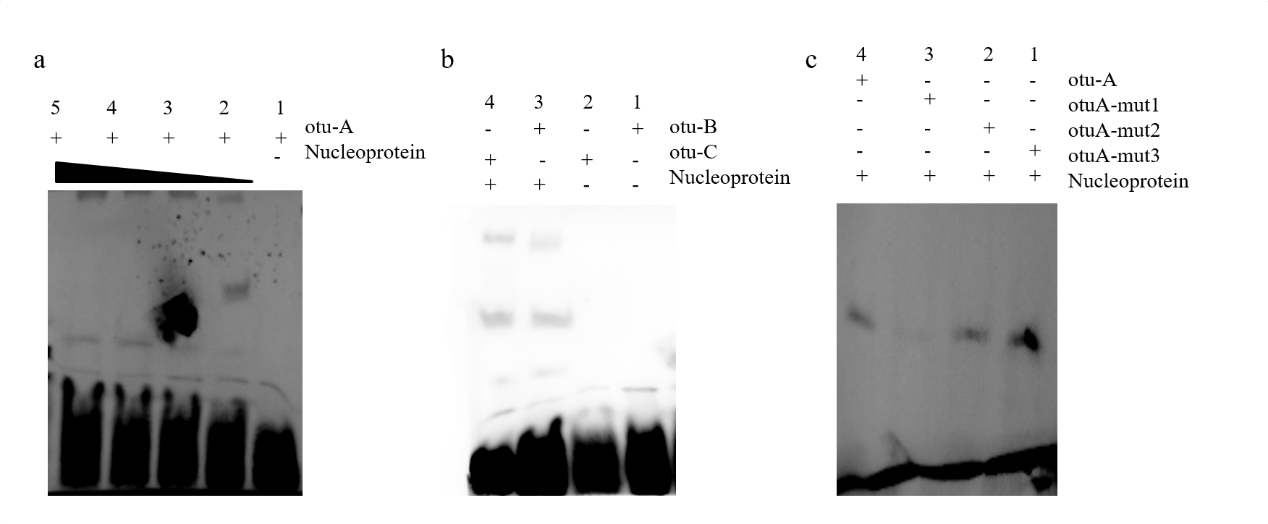
**

Figure S3. The Bmotu promoter could bind to nucleoproteins. (a) analysis of the binding of probe otu-A to different concentrations of nucleoproteins, lane1-5: the final concentration of nucleoproteins is 0, 0.175, 0.35, 0.525, 0.7mg/mL; (b) otu-B and otu-C probes binding to nucleoproteins; (c) otu-A, otuA-mut1, otuA-mut2 and otuA-mut3 binding to the nucleoproteins.

**Table S1. Different combinations of *luc* expression plasmids.**

| Different combinations of BmOVOs | Copies of *luc* expression plasmids (unit: copy) | | | |
| --- | --- | --- | --- | --- |
|  | pFast-potu5-Luc  -ie1-Bmovo1 | pFast-potu5-Luc  -ie1-Bmovo2 | pFast-potu5-Luc  -ie1-Bmovo3 | pFast-potu5-Luc  -ie1-Bmovo4 |
| BmOVO1 | 1×10^11^ | — | — | — |
| BmOVO1+BmOVO2 | 5×10^10^ | 5×10^10^ | — | — |
| BmOVO1+BmOVO3 | 5×10^10^ | — | 5×10^10^ | — |
| BmOVO1+BmOVO4 | 5×10^10^ | — | — | 5×10^10^ |
| BmOVO2 | — | 1×10^11^ | — | — |
| BmOVO2+BmOVO3 | — | 5×10^10^ | 5×10^10^ | — |
| BmOVO2+BmOVO4 | — | 5×10^10^ | — | 5×10^10^ |
| BmOVO3 | — | — | 1×10^11^ | — |
| BmOVO3+BmOVO4 | — | — | 5×10^10^ | 5×10^10^ |
| BmOVO4 | — | — | — | 1×10^11^ |

**Table S2. The plasmids used in this study**

| Name of plasmid | Description |
| --- | --- |
| pIZT/V5-His-STAT | containing the signal transduction and transcription activation factor (*STAT*) gene |
| pIZT/V5-His-DPP | containing the decapentaplegic (*Dpp)* gene, which is member of the transforming growth factor-β (TGF-β family) |
| pIZT/V5-His-Daw | containing dawdle (*Daw*) gene, which is member of the transforming growth factor-β (TGF-β family) |
| pIZT/V5-His-Ror2 | containing the tyrosine kinase-like orphan receptor (*Ror2*) gene ([AK385238.1](https://www.ncbi.nlm.nih.gov/nucleotide/346705453?report=genbank&log$=nuclalign&blast_rank=2&RID=0KFJDG9W01R)), which is a Wnt pathway regulatory factor |
| pIZT/V5-His-BBX-B8 | containing the insulin-like peptide *BBx-B8* |
| pIZT/V5-His-tal | with a full-length cDNA sequence of Tal-like |
| pIZT/V5-His-5A1-4+B | with a sequence containing the 5'-non-coding sequence and the downstream sequence 1A–4A with B |
| pIZT/V5-His-A1-4+B | with a sequence containing A1–A4 with B |
| pIZT/V5-His-B | with a sequence containing B and its downstream non-coding sequence |

**Table S3 The primers used in this paper**

| Primers | Sequences (5’-3’) | Name of DNA fragments |
| --- | --- | --- |
| oie-1 | GGATCCGATTTGCAGTTCGGGACATA | *ie-1* promoter |
| oie-2 | GCGGCCGCAGTCGTTTGGTTGTTCACGAT | *ie-1* promoter |
| Luc-1 | CCCGGGCATGGAAGACGCCAAAAAC | luciferase (*luc*) gene |
| Luc-2 | GGTACCTTACACGGCGATCTTTCCGC | luciferase (*luc*) gene |
| ovo-A1 | GCGGCCGCATGTTAAATGCCGCTGCCGG | *Bmovo-1* |
| ovo-B1 | GCGGCCGCATGTTAAATGCCGCTGCCGG | *Bmovo-2* |
| ovo-C1 | GCGGCCGCATGAGCAGCAACTGGAGCTG | *Bmovo-3* |
| ovo-D1 | GCGGCCGCATGAGCAGCAACTGGACTGC | *Bmovo-4* |
| ovo-A2 | TCTAGATTAATTGTGTACTGGCATGGG | *Bmovos* |
| ovo-T2 | GCGGCCGCATGGACCCGCTCTGTCCCGATTC | *Bmovo*-T2 |
| ovo-T3 | GCGGCCGCATGACAATTCCAACAACACTACCAC | *Bmovo*-T3 |
| ovo-TB1 | GCGGCCGCATGGCCGCTGCCGGTCATGGAC | *Bmovo*-TB1 |
| ovo-T4 | GCGGCCGCATGAATTCATTTAATGCAAACAGCAAC | *Bmovo*-T4 |
| ovo-T5 | GCGGCCGCATGGGATTCAACATGGACTGTG | *Bmovo*-T5 |
| ovo-TB2 | GCGGCCGCATGCAAATGCAACAGCTTCAAATTC | *Bmovo*-TB2 |
| ovo-T1 | GCGGCCGCATGGAGCAGCAACTGGAGCTGCAGGAAGGCCAGGAGTTGCTCTTAAATGCCGCTGCCGGTCATG | *Bmovo-2* with A1 domain |
| Potu2-1 | CCCGGGATGCCGAAAATCTTCTGGAT | *Bmotu* promoters(potu) |
| Potu1 | GGATCCGAGACACTCGAAGCACCACG | potu with control element (CE):  CE1, CE2 and CE3 |
| Potu5 | GGATCCGGTAATGTAACACTATGGAAGTATAC | potu with CE2 and CE3 |
| Potu7 | GGATCCCATGCACCTACGACATGC | potu with CE3 |
| Potu8 | GGATCCTAAAATGTACCGTTGTAACTTCTGCATGCACCTACGACATG | potu with CE1 and CE3 |
| OTU-M2-1 | GGATCCAATGAACCGTTGTAACTTCTGTACATA | potu with mutations at the CE1 |
| OTU-M2-2 | GGATCCAATGGACCGTTGTAACTTCTGTACATA | potu with mutations at the CE1 |
| OTU-M2-3 | GGATCCAATGCACCGTTGTAACTTCTGTACATA | potu with mutations at the CE1 |
| OTU-M3-1 | GGATCCAATGTTCCGTTGTAACTTCTGTACATA | potu with mutations at the CE1 |
| OTU-M3-2 | GGATCCAATGTGCCGTTGTAACTTCTGTACATA | potu with mutations at the CE1 |
| OTU-M3-3 | GGATCCAATGTCCCGTTGTAACTTCTGTACATA | potu with mutations at the CE1 |
| OTU-M11-1 | GGATCCAATGTACCGTTGTTACTTCTGTACATA | potu with mutations at the CE1 |
| OTU-M11-2 | GGATCCAATGTACCGTTGTGACTTCTGTACATA | potu with mutations at the CE1 |
| OTU-M11-3 | GGATCCAATGTACCGTTGTCACTTCTGTACATA | potu with mutations at the CE1 |
| OTU-D2 | GGATCCAATGACCGTTGTAACTTCTGTACATA | potu with deletions at the CE1 |
| OTU-D3 | GGATCCAATGTCCGTTGTAACTTCTGTACATA | potu with deletions at the CE1 |
| OTU-D11 | GGATCCAATGTACCGTTGTACTTCTGTACATA | potu with deletions at the CE1 |
| OTU-D2+3 | GGATCCAATGCCGTTGTAACTTCTGTACATA | potu with deletions at the CE1 |
| OTU-D2+11 | GGATCCAATGACCGTTGTACTTCTGTACATA | potu with deletions at the CE1 |
| OTU-D3+11 | GGATCCAATGTCCGTTGTACTTCTGTACATA | potu with deletions at the CE1 |
| OTU-D2+3+11 | GGATCCAATGCCGTTGTACTTCTGTACATA | potu with deletions at the CE1 |
| Bmovo2-3 | GGATCCATGAACGCAAGACAAAGGGAAG | The sequence encoding four zinc-finger domains of BmOVO |
| Bmovo2-2 | AAGCTTATTGTGTACTGGCATGGG | The sequence encoding four zinc-finger domains of BmOVO |
| ovo1-dsred-F | GGTACC**ATGCCGAAAATCTTCTGGATTAAGAAGCGACTCCATGAGCAGCAACTGGAGCTGCAGGAAGGCCAGGAGTTGCTCGCCAGCAAG**ACTGTGCGCTCCTCCAAGAACGT | *dsRed* gene fused with the N-terminal domain sequence (28 aa) of BmOVO-1 |
| ovo1-dsred-R | TCTAGACTACAGGAACAGGTGGTGGC | *dsRed* gene fused with the N-terminal domain sequence (28 aa) of BmOVO-1 |

The sequence with underline indicates the enzyme sites, and with wave underline indicates the binding site CE1. Base with frame indicates the mutation. In primer ovo1-dsred-F, the double underline was 5’-terminal sequence of dsred gene, the sequence with boldface were the coding sequence of N- terminus 28 residues of BmOVO-1.

**Table S4. Sequence of mutant DNA probe.**

| Probes | Sequences (5’-3’) |
| --- | --- |
| otuA-mutant1 | Biotin-GCCCCTAAAATGCACCGTTGTAACTTCTGT |
|  | Biotin-ACAGAAGTTACAACGGTGCATTTTAGGGGC |
| otuA-mutant2 | Biotin-GCCCCTAAAATGTGCCGTTGTAACTTCTGT |
|  | Biotin-ACAGAAGTTACAACGGCACATTTTAGGGGC |
| otuA-mutant3 | Biotin-GCCCCTAAAATGTACCGTTGTGACTTCTGT |
|  | Biotin-ACAGAAGTCACAACGGTACATTTTAGGGGC |

Sequence of mutant DNA probe. The sequence with underline indicates the predicted binding sites, and base with frame indicates the mutation.
